# Stressful Life Events and Life Satisfaction among Chinese Older Adults: The Role of Coping Styles

**DOI:** 10.3390/healthcare9121620

**Published:** 2021-11-23

**Authors:** Qiang Ren, Chaoxin Jiang, Shan Jiang

**Affiliations:** 1 Department of Sociology, Zhejiang University, Hangzhou 310058, China; renq@zju.edu.cn; 2 Department of Social Welfare and Risk Management, School of Public Affairs, Zhejiang University, Hangzhou 310058, China; chaoxinjiang@zju.edu.cn

**Keywords:** stressful life events, coping styles, life satisfaction, older adults

## Abstract

This study aims to investigate the mediating effect of coping styles in the relationship between stressful life events and life satisfaction among Chinese older adults. To test the hypotheses, cross-sectional data (*n* = 8799) from the 2016 China Longitudinal Aging Social Survey (CLASS) were analyzed by Stata 15/SE in this study. Results indicated that stressful life events directly affected life satisfaction. Moreover, the association between stressful life events and life satisfaction was partially mediated by coping styles. This study had implications for the current body of knowledge and provided empirical evidence for social work practice and social policy.

## 1. Introduction

People’s life expectancies have been steadily increasing as medical treatment has improved, posing a global challenge of an aging population. China is experiencing the world’s largest and fastest aging process. According to the National Bureau of Statistics of China, there were 240.9 million people aged 60 and over in China at the end of 2017, accounting for 17.3% of the total population. Even though people face various stressful life events throughout their lives, later life can be a particularly stressful stage, in which older people face interpersonal, financial, and health-related life events, specifically including retirement, physical illness, and bereavement [1]. Stressful life events are generally viewed as predictors negatively impacting older adults’ physical, psychological, and cognitive functioning [2,3].

Aging is unavoidable for everyone. The concept of successful aging has been proposed to describe later life as a condition of continuous health and vigor [4]. Life satisfaction refers to a subjective evaluation of life as a whole, and has been perceived as one of the crucial indicators of successful aging and well-being of older people [4,5,6]. Life satisfaction is positively associated with quality of life, physical health and emotional conditions [7,8,9]. Accordingly, it is critical to achieve a better understanding of life satisfaction and its influencing factors and mechanisms.

Some studies have investigated the relationships between stressful life events and successful aging among older adults, but existing studies mainly focused on the impact of stressful life events on psychopathological outcomes, such as depression, increased risk of suicide, and sense of loneliness [10,11]. Little research has been conducted to investigate the effect of stressful life events on life satisfaction as well as its underlying mediating mechanisms. In such a context, further study is needed to fill this research gap by investigating the effect of stressful life events on life satisfaction in a sample of Chinese older adults.

## 2. Literature Review

Stressful life events refer to the events that interfere with daily life, such as bereavement, interpersonal conflicts, severe illness and poor adaptation [12,13]. Additionally, a large scope of studies has demonstrated that being exposed to stressful life events would result in high depression [10], more anxiety [3] and low life satisfaction [13]. To be specific, Hannaford et al., found that stressful life events would reduce life satisfaction among Scottish community-based older adults [3]. Another study conducted in the United States also indicated that lifetime trauma exposure (e.g., death of spouse or children) was negatively related to older adults’ life satisfaction [14]. Despite that the direct impact of stressful life events has been examined, the mediating mechanism underlying such association was not fully understood.

The stress process model provided a framework to illustrate the mechanisms that connect exposure to stressors and health outcomes of individuals [15]. This model suggested that the association between stressful events and individual outcomes could be mediated or moderated by internal personal factors and external social factors [15]. According to Folkman, coping styles can be defined as “*one’s cognitive and behavioral efforts to handle the demands resulting from the stressful person–environment relationship”* [16]. Active coping refers to the initiative to seek help and problem solutions [17], while poor coping includes escaping from stressors and other maladaptive coping skills [18,19]. It has been generally acknowledged that coping styles played a significant role in individuals’ psychological health [20,21,22]. Active coping styles had a positive impact on well-being and life satisfaction [23,24,25], meanwhile it could also reduce distress, depressive symptoms and protect mental health [26,27].

In addition, stressful life events, as a social contextual factor, would affect how people cope. Cognitive stress theory points out that coping styles have an impact on individuals’ well-being by affecting how they respond to stress exposure [28]. In other words, frequent exposure to stressful events can affect the way older people manage their behaviors and emotions [29,30]. For instance, Undheim and Sund found that interpersonal conflict was a predictor of coping strategies based on participants in Norway [31]. Another longitudinal research conducted by Steeger et al., also supported that facing stressors was associated with more negative coping [32].

**Figure 1:**
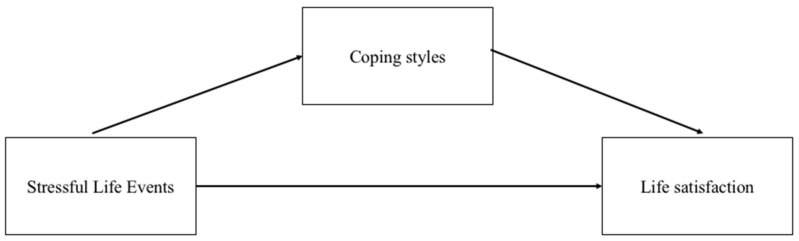
Illustration of the theoretical framework. Note. For simplicity of presentation, control variables are not shown.

In summary, based on the above theoretical and empirical evidence, copying styles can serve as a potential mediator between stressful life events and older adults’ life satisfaction. However, most of the existing studies focused on the direct effect of stressful life events or coping strategies on older people’s life satisfaction. A rare study explored the mediating effect of coping styles between stressful life events and life satisfaction among older adults in the context of China. To fill in the above research gap, this study aims to explore the mediating role of coping styles in the association between stressful life events and older adults’ life satisfaction (See Figure 1). This study proposed the following research hypotheses:

**Hypothesis** **1.** *More stressful life events are associated with a lower level of life satisfaction.*

**Hypothesis** **2.** *More stressful events are associated with a lower level of active coping styles, which in turn, is associated with a lower level of life satisfaction.*

## 3. Methods

### 3.1. Participants

Data in this research were from the China Longitudinal Aging Social Survey (CLASS) in 2016, a large-scale national social survey carried out by the National Survey Research Center of Renmin University of China. A stratified multi-stage probability sampling method was adopted by CLASS 2016 and total of 8799 valid samples of the elderly aged 60 and over for data analysis were finally obtained in this study. The sample composition was as follows: Males accounted for 51.9%, and females accounted for 48.1%. The average age of the samples was 69.57 (S.D. = 7.25). The older people who had spouse were 73.2%, whereas those who did not have spouse (e.g., single, divorce or bereave) were 26.8%. The proportion of older people with chronic diseases was 55.8%, while older adults without chronic diseases accounted for 44.2%. More detailed descriptions of socio-economic conditions were presented in Table 1.

### 3.2. Measurement

Stressful life events: Participants of CLASS 2016 were asked whether they had encountered the following events in the past year: severe illness, natural disaster, death of spouse, death of children, death of other relatives and friends, property loss, serious illness of family, conflict with relatives and friends, change of residence, and accident. Each question was coded as 0 = not happened, 1 = happened. We amounted the scores for these ten items to evaluate stressful life events, with a higher score demonstrating that they faced more stressful life events.

Coping styles: This study used the adapted version of the Simplified Coping Style Questionnaire [18] to assess coping styles. In CLASS 2016, SCSQ contains six questions to measure how old people cope with stressors in daily life. The scale has six items, including “learning from others”, “changing thinking”, “talking with others”, “accepting the reality”, “relying on others” and “trying to forget”. A 4-point Likert scale ranging from “1 = absolutely not adopted” to “4 = usually adopted” was adopted to access the level in each coping style. The negative items were recoded, and the scores of six items were summed. A higher score indicates a higher level of active coping adopted by older adults. In previous studies, SCSQ showed good reliability and validity [18,33]. The Cronbach’s for coping styles in this study was 0.747, which showed acceptable reliability.

Life satisfaction: The dependent variable of this research is life satisfaction. In CLASS 2016, life satisfaction was evaluated by a single item, which is “In general, are you satisfied with your life?”. This item was measured on a 5-point Likert scale from “1 = very dissatisfied” to “5 = very satisfied”. Especially for older people, the single-item measurement of life satisfaction has been used widely and is considered reliable [34,35].

Socio-demographic characteristics: The socio-economic conditions, including gender (1 = male, 0 = female), age, spouse (1 = yes, 0 = no), religion (1 = yes, 0 = no), chronic illness (1 = yes, 0 = no) and residence (1 = local, 0 = non-local), were controlled in the statistic model.

### 3.3. Data Analysis

In order to test the hypothetical theoretical model proposed in our study, the mediating effect of coping styles was validated utilizing Stata 15/SE (StataCorp LLC, College Station, TX, USA). The key variables with significant missing data were dealt with list-wise deletion. Normality and outliers’ analyses were conducted before formal analysis. The significance of indirect effect through coping styles was assessed by bootstrapping methods (2000 iterations for the present study) using 95% confidence intervals. The effect was considered significant if there is not a ‘zero’ value between the upper and lower bounds of the confidence intervals [36].

## 4. Results

### 4.1. Descriptive Analyses

As presented in Table 2, the means, standard deviations, and Pearson’s correlations of stressful life events, coping styles, and life satisfaction were evaluated. To be specific, life satisfaction was negatively correlated with stressful life events (*r* = −0.127, *p* < 0.001), and positively correlated with coping styles (*r* = 0.073, *p* < 0.001).

### 4.2. Testing of Hypothesis

We hypothesized that coping styles would mediate the relationship between stressful life events and life satisfaction of older adults. In order to validate our assumptions, the parameters for two regression models were estimated and the detailed results of standardized path coefficients were presented in Table 3.

As expected, the direct effect of stressful life events on geriatric life satisfaction was significant (*β* = −0.118, *p* < 0.001). Moreover, stressful life events were negatively related to coping styles (*β* = −0.036, *p* < 0.001). Furthermore, coping styles were positively associated with life satisfaction (*β* = 0.063, *p* < 0.001). The bootstrap test indicated that the indirect effect of stressful life events on life satisfaction via coping styles were significant (*β* = −0.004, 95% CI = [−0.007 −0.002]). Accordingly, the assumption of the mediating effect of coping styles in our study was supported. In other words, stressful life events decreased active coping styles, which in turn reduced the degree of life satisfaction among older people.

## 5. Discussion

Using a nationally representative sample of older adults in China, this study validated the relationship between stressful life events, coping styles, and life satisfaction among Chinese older people. All research hypotheses in this study were supported. As expected, more stressful life events would result in a lower level of coping styles, thereby decreasing life satisfaction in older people. The results were interpreted and discussed as follows.

As predicted, and in accordance with previous literature conducted in Western countries [3,14], more frequent exposure to stressful life events could lead to a lower level of life satisfaction among Chinese older adults. Moreover, this study supported the mediating role of coping styles. This finding was in line with the prior research, demonstrating that more stressful events were associated with a lower level of coping styles [31,32], which in turn, resulted in a lower level of life satisfaction [23,24,25]. To explain, effective coping styles need the utilization of cognitive abilities and behavioral resources [37]. However, frequent stressful life events will lead these resources to be depleted [38]. Once older people’s ability to master the environment declines and cannot to take effective emotional adjustment, their life satisfaction may decrease [10,37]. These findings echoed the stress process model and provided cross-cultural empirical evidence in the Chinese social context.

To the best of our knowledge, this study is among the first to examine the association between stressful life events, coping styles, and life satisfaction in a nationally representative sample of Chinese older adults. This study is highlighted by its distinction from prior literature using regional samples. In addition, this study examines the mediating role of coping styles between stressful life events and life satisfaction, which is understudied in previous research. This study expanded the knowledge in this field and deepened our understanding of how the internal individual factor (coping styles) influences the path from stressors to the well-being of older adults by providing robust empirical evidence from China. This study also provides empirical support for the stress process model to help us better understand the mechanism involved in successful aging. The findings of this study can serve as a foundation for future research on successful aging.

## 6. Implication

On the basis of the empirical findings, being aware of the harmfulness of stress exposure among older adults and providing effective intervention programs are significant. Even though the need for psychological assistance for older adults is rapidly increasing, there are still few interventions designed to induce older people to confront stressful life events [39]. According to the previous studies, exposure-based therapies are supported as one of the effective treatments that help older adults to cope with stressful events [40]. For example, exposure therapy via the Autobiographical Memory Questionnaire (AMQ) was empirically supported by Boals et al. [41]. On the one hand, it can save resources and time, and on the other hand, it helps to encourage older people to have a ‘self-help’ mentality and reduce the stigma. Although this intervention was conducted in western societies, it may be applied and replicated in China as well. In addition, given the negative consequences of stressful events, this study also calls on the government to pay attention to older adults and lessen the risk and stress factors in society on the policy level [42].

Moreover, this study highlights the importance of coping styles due to its mediating effect. To be specific, as age increases, not only the use of positive coping styles decreases, but the use of negative coping styles increases [43]. Therefore, interventions that aim at improving the coping styles of older people are highly encouraged. Many gerontologists believe that reminiscence therapy can promote successful aging and enhance the positive coping styles, such as seeking social support and emotional control [43]. In particular, instrumental reminiscence helps to make stressors manageable and enable older adults to adapt to the stressful situation. Furthermore, life satisfaction in later life is an important indicator of active aging in older adults and has received lots of attention in recent decades [35]. In previous studies, meditation interventions, such as concentration, centering and stillness, have been shown to be effective in improving life satisfaction in Asian older adults [44]. In the Chinese context, clinical psychologists and geriatric social workers can draw on these intervention programs to improve the life satisfaction of Chinese older adults. Meanwhile, the government should also allocate more resources to older people and provide support in various aspects so that they can have a better life satisfaction.

## 7. Limitation and Future Direction

The limitations of this study should be noted. First, the cross-sectional research method used in this study could only verify the relationship among stressful life events, coping styles, and life satisfaction from the theoretical level, but cannot determine the causal relationships between the above concepts. Therefore, the results of this study need to be further verified by future longitudinal studies. Secondly, life satisfaction in this study is measured with a single item due to the secondary data limits. Future studies are encouraged to use well-validated scales to comprehensively assess this concept, although single-dimensional measurement of life satisfaction is confirmed to be effective [34,35].

## 8. Conclusions

Based on a national sample of Chinese older adults, this study established a new conceptual framework to explain the mechanism among stressful life events, coping styles, and geriatric life satisfaction in the Chinese context. This study found that stressful life events directly affected geriatric life satisfaction; and coping styles played a mediating role in the relationship between stressful life events and life satisfaction.

## Figures and Tables

**Table 1:** Demographic characteristics of participants (*n* = 8799).

Variable	Category	Frequency (*n*)	Percentage (%)
Gender	Female	4231	48.09
	Male	4568	51.91
Age	Range: 60–103	M = 69.57	SD = 7.25
Spouse	Yes	6439	73.18
	No	2360	26.82
Religion	Yes	763	8.67
	No	8036	91.33
Chronic Illness	Yes	4910	55.80
	No	3889	44.20
Residence	Local	8499	96.59
	Non-local	300	3.41

**Table 2:** Descriptive statistics and bivariate correlations of key variables (*n* = 8799).

Variable	Range	M	SD	1	2	3	4
1. Stressful life events	0–4	0.185	0.476	1000			
2. Coping styles	6–24	16.137	2.251	−0.040 ***	1000		
3. Life satisfaction	1–5	3.847	0.796	−0.127 ***	0.073 ***	1000	

Note. *** *p* < 0.001.

**Table 3:** Test of the mediation model (*n* = 8799).

Variable	Model 1 (Coping Styles)			Model 2 (Life Satisfaction)		
	*β*	*z*	*p*	*β*	*z*	*p*
Gender	0.004	0.37	0.708	−0.012	−1.15	0.251
Age	−0.041	−3.64	<0.001	0.046	4.19	<0.001
Spouse	0.024	2.10	0.036	0.070	6.21	<0.001
Religion	−0.003	−0.24	0.807	0.024	2.31	0.021
Chronic illness	−0.044	−4.07	<0.001	−0.095	−8.99	<0.001
Residence	0.008	0.76	0.450	−0.006	−0.53	0.598
Stressful life events	−0.036	−3.42	0.001	−0.118	−11.29	<0.001
Coping styles				0.063	6.00	<0.001

## Data Availability

Data sharing not applicable.
